# Magnolol Inhibits Osteoclast Differentiation via Suppression of RANKL Expression

**DOI:** 10.3390/molecules23071598

**Published:** 2018-07-02

**Authors:** Youn-Hwan Hwang, Taesoo Kim, Rajeong Kim, Hyunil Ha

**Affiliations:** Herbal Medicine Research Division, Korea Institute of Oriental Medicine, Daejeon 34054, Korea; hyhhwang@kiom.re.kr (Y.-H.H.); xotn91@kiom.re.kr (T.K.); younme1112@nate.com (R.K.)

**Keywords:** magnolol, osteoblast, osteoclast, interleukin-1, prostaglandin E_2_

## Abstract

Magnolol, a compound from the traditional Korean herb *Magnolia* sp., has been exhaustively investigated as a therapeutic agent against several diseases including systemic and local inflammation. We examined the effects of magnolol on osteoclastic differentiation associated with inflammation. Magnolol markedly reduced interleukin (IL)-1-induced osteoclast formation in co-cultures of murine osteoblasts and bone marrow cells, whereas it had no effect on receptor activator of nuclear factor-κB ligand (RANKL)-induced osteoclast formation in bone marrow macrophage cultures. In osteoblasts, magnolol markedly inhibited both the up-regulation of RANKL expression and the production of prostaglandin E_2_ (PGE_2_) in response to IL-1 treatment. Addition of exogenous PGE_2_ reversed the inhibitory effects of magnolol on IL-1-induced RANKL expression in osteoblasts and osteoclast formation in co-cultures. Magnolol inhibited IL-1-induced PGE_2_ production, at least in part by suppressing cyclooxygenase-2 (COX-2) expression. Taken together, these results demonstrate that magnolol inhibits IL-1-induced RANKL expression in osteoblasts through suppression of COX-2 expression and PGE_2_ production, resulting in inhibition of osteoclast differentiation in co-cultures.

## 1. Introduction

Osteoclasts are multinucleated, bone-resorbing cells produced via differentiation from monocyte/macrophage lineage cells. Receptor activator of nuclear factor (NF)-κB (RANK) ligand (RANKL), expressed on osteoclast-supporting cells including osteoblasts and stromal cells, is an essential cytokine that induces osteoclast differentiation and activation by binding to its receptor RANK expressed on osteoclast precursors and osteoclasts [1]. Because the RANKL–RANK axis is crucially involved in pathological bone destruction and normal bone remodeling, inhibition of this axis can be an attractive therapeutic strategy to reduce excessive bone loss [1].

Chronic inflammation, a key mediator of local and systemic bone loss, is linked to an increased risk of osteoporotic fracture in patients with arthritis, periodontitis, and inflammatory bowel diseases, and in healthy individuals with low-grade inflammation [2]. Among pro-inflammatory mediators, interleukin (IL)-1 plays a key role in bone destruction under pathological conditions such as rheumatoid arthritis and osteoporosis [3]. At the cellular levels, IL-1 can directly stimulate osteoclastic bone resorption through multiple mechanisms including promotion of osteoclast precursor fusion, enhancement of bone-resorbing activity of osteoclasts, and prolongation of osteoclast survival [4,5]. In addition, IL-1 also indirectly stimulates osteoclast differentiation by up-regulating the expression of RANKL and down-regulating osteoprotegerin (OPG), the decoy receptor for RANKL, in osteoblasts [6].

**Figure 1:**
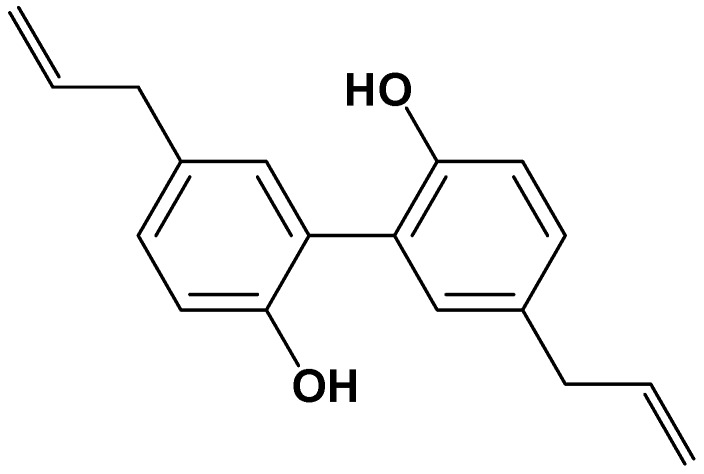
Chemical structure of magnolol.

Magnolol (5,5′-diallyl-2,2′-dihydroxybiphenyl, Figure 1), the primary bioactive compound in *Magnolia obovate* and *M*. *officinalis*, has a broad spectrum of biological activities including anti-inflammatory, antioxidant, antimicrobial, and antitumor effects [7,8,9]. This compound has also been shown to exhibit beneficial effects in various experimental models of inflammatory diseases including mastitis, arthritis, sepsis-induced intestinal dysmotility, and periodontitis [7,8,10]. It was reported that magnolol inhibits alveolar bone loss in ligature-induced periodontitis in rats with reduced gingival inflammation, RANKL expression, and osteoclast numbers [8]. However, it is still unclear whether and how magnolol affects osteoclast differentiation. Magnolol (5–20 μm) has been shown to inhibit RANKL-induced osteoclast differentiation of mouse macrophage-like RAW264.7 cells [8,11]. In contrast, we previously showed that magnolol (up to 20 μm) does not affect RANKL-induced osteoclast differentiation of its precursors, mouse primary bone marrow-derived macrophages (BMMs) [12]. In the present study, we aimed to investigate the effects of magnolol on osteoclast differentiation associated with inflammation using a co-culture system comprising mouse osteoblasts and bone marrow cells with the pro-inflammatory cytokine IL-1.

## 2. Results and Discussion

### 2.1. Magnolol Inhibits IL-1-Induced Osteoclast Differentiation in Co-Cultures

**Figure 2:**
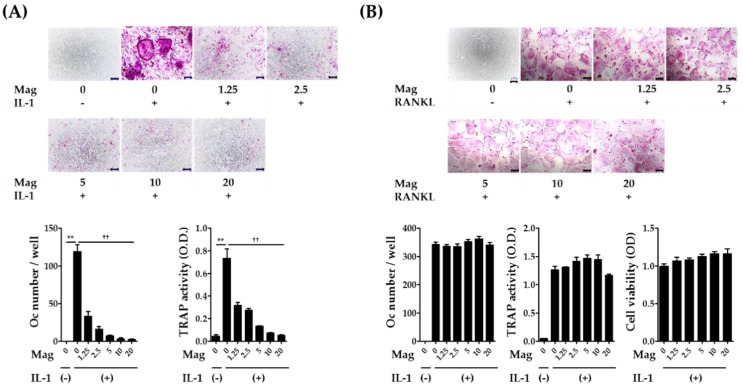
Magnolol inhibits osteoclast formation in co-cultures treated with IL-1. (**A**) Mouse primary osteoblasts and bone marrow cells were co-cultured in the presence of IL-1 (10 ng/mL) for seven days. Magnolol (Mag, 1.25–20 μm) was pretreated for 1 h prior to IL-1 treatment. After fixation and TRAP staining, TRAP-positive multinucleated giant cells (≥three nuclei, ≥50 μm in diameter) were counted as osteoclasts. (**B**) BMMs were incubated in the presence or absence of M-CSF (30 ng/mL), RANKL (100 ng/mL), and Mag for four days. The number of osteoclasts was counted. Cell viability was determined via Cell Counting Kit-8 assay. Scale bar, 100 μm. ** *p* < 0.01 vs. IL-1-untreated control. ^††^ *p* < 0.01 vs. magnolol-untreated control.

Various osteotropic factors such as IL-1, parathyroid hormone, 1,25-dihydroxyvitamin D3, and prostaglandin E_2_ (PGE_2_) induce RANKL expression on osteoblasts leading to the differentiation of osteoclast precursors into osteoclasts in a co-culture system comprising osteoblasts and osteoclast precursors [3]. Among these factors, IL-1 has been shown to mediate pathological bone destruction in a variety of inflammatory conditions including rheumatoid arthritis and periodontitis [3]. Therefore, we selected IL-1 as an osteoblast stimulator for the induction of osteoclast differentiation. The co-cultures treated with IL-1 for seven days showed increased tartrate-resistant acid phosphatase (TRAP) activity, a maker of osteoclast differentiation, and osteoclast formation that was dose dependently inhibited by magnolol (Figure 2A). Almost complete inhibition of osteoclast formation was observed at a concentration of 10 μm. Subsequently, BMM cultures under the treatment of macrophage colony-stimulating factor (M-CSF) and RANKL were used to clarify the inhibitory effects of magnolol on osteoclast formation. There were no anti-osteoclastogenic or cytotoxic effects of magnolol in BMM cultures (Figure 2B). These results suggest that magnolol inhibits osteoclast formation via affecting the ability of osteoblasts to support osteoclast differentiation.

### 2.2. Magnolol Decreases the Expression of RANKL in IL-1-Stimulated Osteoblasts

**Figure 3:**
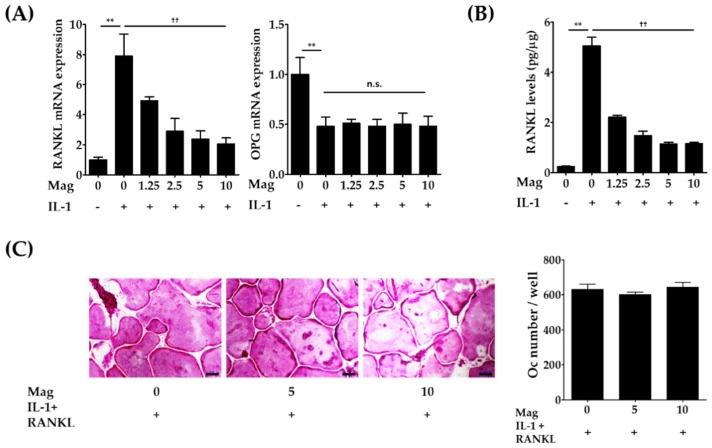
Magnolol interferes with IL-1-induced RANKL expression in osteoblasts. (**A**) Primary osteoblasts were incubated with or without IL-1 (10 ng/mL) or magnolol (Mag, 1.25–10 μm) for 24 h. Gene expression levels of RANKL and OPG in osteoblasts were analyzed via real-time quantitative PCR. (**B**) The protein expression levels of RANKL in osteoblast lysates were determined using an ELISA kit. (**C**) Primary osteoblasts and bone marrow cells were co-cultured with or without magnolol (5 and 10 μm), IL-1 (10 ng/mL), and RANKL (100 ng/mL) for five days. The number of osteoclasts was counted. Scale bar, 100 μm. ** *p* < 0.01 vs. IL-1-untreated control. ^††^ *p* < 0.01 vs. magnolol-untreated control. n.s., no significance.

Because IL-1 has been shown to increase RANKL expression and decrease expression of its decoy receptor OPG on osteoblasts [6,13], we next investigated whether magnolol might modulate RANKL and OPG expression in osteoblasts through real-time polymerase chain reaction (PCR) analysis and enzyme-linked immunosorbent assay (ELISA). Magnolol (1.25–10 μm) significantly inhibited IL-induced RANKL mRNA levels in a dose-dependent manner (*p* < 0.01, Figure 3A); thereby, excess production of RANKL was also markedly reduced by magnolol (*p* < 0.01, Figure 3B). In contrast to RANKL expression, there was no change in IL-1-induced reduction of OPG mRNA levels following magnolol treatment. These results are in good agreement with those of a previous in vivo study showing that magnolol inhibits ligature-induced up-regulation of RANKL expression without affecting down-regulation of OPG expression in gingival tissues [8]. Next, we examined whether the inhibitory effect of magnolol could be suppressed by RANKL supplement. As shown in Figure 3C, the exogenous addition of RANKL significantly elevated IL-1-induced osteoclast formation and reversed the inhibitory effect of magnolol on IL-1-induced osteoclast formation in co-cultures, confirming that magnolol has no direct inhibitory effects on RANKL-induced differentiation of osteoclast precursors into osteoclasts. Thus, our results suggest that the anti-osteoclastogenic effect of magnolol in co-cultures was mainly due to the inhibition of IL-1-induced up-regulation of RANKL expression.

### 2.3. Magnolol Reduces IL-1-Induced PGE_2_ Production by Inhibiting Cyclooxygenase-2 (COX-2) Expression

**Figure 4:**
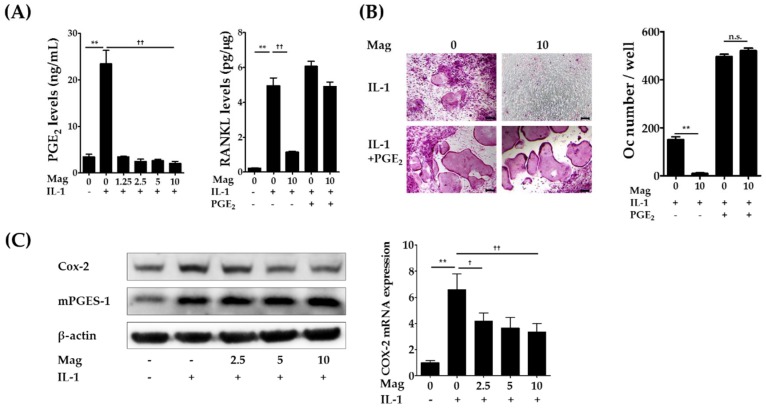
Magnolol suppresses IL-1-induced PGE_2_ production by inhibiting COX-2 expression in osteoblasts. (**A**) Osteoblasts were incubated with or without IL-1 (10 ng/mL) or magnolol (Mag, 1.25–10 μm) for 24 h. The levels of RANKL in cell lysates and PGE_2_ in culture media were determined. (**B**) Osteoblasts and bone marrow cells were co-cultured in the presence of IL-1 (10 ng/mL) with or without Mag (10 μm) and PGE_2_ (100 nm) for seven days. Cells were stained for TRAP, and the number of osteoclasts was counted. (**C**) Osteoblasts were treated with or without IL-1 (10 ng/mL) and magnolol (2.5–10 μm) for 24 h. The protein levels of COX-2 and mPGES-1 were determined via Western blot (left). COX-2 mRNA levels were analyzed using real-time PCR (right). Scale bar, 100 μm. ** *p* < 0.01 vs. IL-1-untreated control. ^†^ *p* < 0.01 and ^††^ *p* < 0.01 vs. magnolol-untreated control. n.s., no significance.

We previously showed that NS-398, a specific inhibitor of COX-2, inhibits IL-1-induced RANKL expression in osteoblasts and osteoclast formation in cocultures, and the addition of either PGE_2_ or RANKL reversed the inhibitory effects of NS-398 on osteoclast formation in cocultures, suggesting that PGE_2_ mediates IL-1-induced osteoclast formation in cocultures mainly by stimulating RANKL expression in osteoblasts [6]. Therefore, we next examined whether PGE_2_ production is involved in the inhibitory effects of magnolol on RANKL expression and osteoclast formation. Magnolol (1.25–10 μm) significantly inhibited IL-1-induced PGE_2_ production in osteoblasts (Figure 4A, left panel). The addition of exogenous PGE_2_ fully restored IL-1-induced RANKL expression in osteoblasts (Figure 4A, right panel) and osteoclast formation in cocultures (Figure 4B) inhibited by magnolol at the highest concentration (10 μm). These findings indicate that magnolol-induced suppression of PGE_2_ production contributes to its anti-osteoclastogenic effects.

PGE_2_ is synthesized from membrane phospholipids via the sequential action of the three enzymes phospholipase A2 (PLA2), COX, and PGE synthase (PGES) [14]. Previous studies have shown that cytosolic PLA2α, COX-2, and microsomal PGES-1 (mPGES-1) are key isoenzymes in PGE_2_ synthesis induced by IL-1 and LPS in osteoblasts and stromal cells [6,15,16], and the expression of COX-2 and mPGES-1 in osteoblasts is markedly enhanced following treatment with IL-1 [6,15]. Therefore, we examined the effects of magnolol on the expression of COX-2 and mPGES-1 in osteoblasts. IL-1 increased the protein expression of COX-2 and mPGES-1, and magnolol suppressed the up-regulation of COX-2 but not of mPGES-1 (Figure 4C). Magnolol also inhibited IL-1-induced COX-2 mRNA levels. These results indicate that magnolol suppresses excessive production of PGE_2_ in IL-1-stimulated osteoblasts, at least in part by inhibiting IL-1-induced COX-2 mRNA levels. Previous studies have shown that magnolol inhibits IL-1-induced COX-2 mRNA expression via inhibition of NF-κB and mitogen-activated protein kinase activation in fibroblast-like synoviocytes [10]. Recently, magnolol was also found to inhibit LPS-induced NF-κB activation via PPARγ induction [17]. However, the precise molecular mechanisms by which magnolol inhibits IL-1-inducd COX-2 mRNA levels in osteoblasts remain to be elucidated.

In the present study, we showed that magnolol indirectly inhibits osteoclast differentiation via suppression of PGE_2_ synthesis and subsequent RANKL expression in osteoblasts treated with IL-1 without directly affecting osteoclast precursors. Apart from the anti-osteoclastogenic effect, magnolol has also been shown to stimulate osteoblast proliferation and differentiation [18]. Thus, these findings suggest that magnolol might have potential in the prevention and treatment of bone disease–associated inflammation.

## 3. Materials and Methods 

### 3.1. Reagents

Magnolol was obtained from Sigma-Aldrich (St. Louis, MO, USA). Alpha-modified minimal essential medium (α-MEM) and fetal bovine serum (FBS) were purchased from Thermo Fisher Scientific Inc. (Rockford, IL, USA). Recombinant IL-1α was obtained from PeproTech (Rocky Hill, NJ, USA). Recombinant M-CSF was kindly provided by Dr. Yongwon Choi (University of Pennsylvania School of Medicine, Philadelphia, PA, USA). Recombinant soluble RANKL was prepared as described previously [19].

### 3.2. Cell Preparation

The animal experiments were approved by the Institutional Animal Care and Use Committee of Korea Institute of Oriental Medicine (permission numbers: 15-057 and 15-058, Daejeon, Korea). Mice were obtained from Samtako (Osan, Korea). Primary osteoblasts were isolated from calvariae of newborn Institute of Cancer Research (ICR) mice, and bone marrow cells and BMMs were isolated from femurs of ICR mice (5–7 weeks old, male) as reported previously [20].

### 3.3. Osteoclast Formation Assays

Primary osteoblasts (2.5 × 10^4^ cells) and bone marrow cells (3 × 10^5^ cells) were co-cultured for five or seven days in α-MEM containing 10% FBS in 48-well tissue culture plates. Co-cultures were incubated in the presence of IL-1 (10 ng/mL), RANKL (50 ng/mL), or PGE_2_ (100 nm) during all experiments. Magnolol was added 1 h before treatment with IL-1, RANKL, or PGE_2_. For osteoclast formation assay in BMM cultures, BMMs (1 × 10^4^ cells/well in a 96-well plate) were cultured for four days in the presence of M-CSF (30 ng/mL) and RANKL (100 ng/mL) with or without magnolol. TRAP assay was performed per the method previously described [19]. TRAP-positive multinucleated (≥three nuclei) cells larger than 50 μm in diameter were considered osteoclasts. Cell viability of BMMs was determined using Cell Counting Kit-8 (Dojindo Molecular Technologies Inc., Rockville, MD, USA) after being cultured with M-CSF and magnolol for two days.

### 3.4. RANKL, OPG, and PGE_2_ Productions in Primary Murine Osteoblasts

In the presence or absence of IL-1 (10 ng/mL), primary osteoblasts (3 × 10^4^ cells) were cultured for 24 h in 12-well culture plates, and magnolol (1.25–10 μm) was added 1 h prior to IL-1 treatment. After incubation, the concentrations of RANKL in primary osteoblast lysates and OPG in the culture media were determined using the corresponding ELISA kits (R&D Systems, Minneapolis, MN, USA) following the manufacturer's instructions. PGE_2_ levels in the culture supernatants were determined using an enzyme immunoassay kit (Cayman Chemicals, Ann Arbor, MI, USA).

### 3.5. Real-Time Quantitative PCR

Following the manufacturer’s instructions, total RNA was extracted and cDNA prepared using the RNeasy kit (Qiagen, Hilden, Germany) and High-Capacity cDNA Reverse Transcription Kit (ABI, Waltham, MA, USA), respectively. Real-time quantitative PCR was performed using TaqMan probes (Thermo Scientific, Rockford, IL, USA) and TaqMan Universal Master Mix in an ABI 7500 Real-Time PCR System. Gene expression of RANKL (Mm0041908_m1), OPG (Mm00435452_m1), and COX-2 (Mm00478374_m1) in osteoblasts were analyzed, and all gene expression experiments were conducted in triplicate. Relative expression was calculated using the ΔΔCt method and the 18S ribosomal gene (Hs99999901_s1) to normalize mRNA expression levels.

### 3.6. Western Blot Assay

Cell lysates were prepared using RIPA lysis buffer (Millipore, MA, USA) with protease and phosphatase inhibitors (Roche Applied Science, Indianapolis, IL, USA). Protein content was measured using a bicinchoninic acid assay kit (Thermo Scientific). Total protein (40 μg) was separated using 12.5% SDS-PAGE gel electrophoresis and electrotransferred to a polyvinylidene fluoride membrane. After blocking with 5% bovine serum albumin, membranes were incubated overnight at 4 °C with primary antibodies at the following concentrations: COX-2 (1:1000, BD Biosciences, Heidelberg, Germany), mPGES-1 (1:500, Cayman Chemicals), and β-actin (1:1000, Santa Cruz Biotechnology, Santa Cruz, CA, USA). The secondary antibodies (1:5000) goat anti-mouse IgG-HRP (Santa Cruz Biotechnology) and goat anti-rabbit IgG-HRP (Santa Cruz Biotechnology) were used for blotting. Chemiluminescent signals were generated via a chemiluminescence reagent (Thermo Scientific) and captured using a ChemiDoc imaging system (Bio-Rad Laboratories, Hercules, CA, USA).

### 3.7. Statistical Analysis

All data are shown as mean ± SD. Experiments were repeated three or four times, and results from one representative experiment are shown. Statistical differences were analyzed using Student’s *t*-test with the software Prism version 5.0. A value of *p* < 0.05 was considered statistically significant.

## 4. Conclusions

In the present study, we investigated the effects of magnolol on osteoclast differentiation. We show that magnolol prevents IL-1-induced osteoclast formation through suppression of RANKL expression by inhibiting COX-2 expression and PGE_2_ synthesis, suggesting beneficial effects of magnolol against several PGE_2_-mediated diseases including inflammatory bone loss. Further investigations are necessary to elucidate the effectiveness and precise mechanism of magnolol action in the treatment and prevention of postmenopausal osteoporosis and bone disorders related to inflammation.
